# Single-cell insights into hepatic fibroblasts and stellate cells: heterogeneity in matrix remodelling in development, health, and disease

**DOI:** 10.1038/s44355-026-00075-x

**Published:** 2026-07-22

**Authors:** Sara Campinoti, Kavitha Kirubendran, Lai Wei, Maya Medic, Omkar Pravin Joshi, Luca Urbani

**Affiliations:** Roger Williams Institute of Liver Studies, School of Immunology & Microbial Sciences, Faculty of Life Sciences and Medicine, King’s College London, Foundation for Liver Research and King’s College Hospital, London, UK

**Keywords:** Cell biology, Computational biology and bioinformatics, Developmental biology

## Abstract

Hepatic fibroblasts and hepatic stellate cells (HSCs) are dynamic regulators of liver development, homeostasis, and disease beyond structural roles. Advances in single-cell transcriptomics have provided new evidence on their heterogeneity, plasticity, and diverse functions across developmental and pathological contexts. This review synthesises contribution from single-cell and single-nuclei RNA sequencing studies to the understanding of the ontogeny, transcriptional diversity, and roles of hepatic stromal populations in foetal and adult liver. We highlight distinct fibroblast and HSC subpopulations identified across development and disease, characterised by extracellular matrix programmes and markers associated with shaping the hepatic microenvironment. We further examine their activation in chronic liver disease and reprogramming into cancer-associated fibroblasts in hepatobiliary cancers, and discuss how single-cell approaches are contributing to the re-evaluation of stromal plasticity by revealing cell-state transitions and informing in vitro modelling and targeted therapies.

## Introduction

Fibroblasts are the main cell type in the stroma, organ’s fibrous connective tissues, and, alongside endothelial and blood cells, are the most common cells in the human body^1,2^. Fibroblasts were described by German pathologist Rudolf Virchow in 1858 who defined them as “spindle-shaped cells of the connective tissue^”^^3^. The term “fibroblast” has been in use for more than 100 years. The “-blast” suffix refers to morphological hallmarks of active protein synthesis, as it was soon identified that those cells were responsible for extracellular matrix (ECM) turnover in steady-state, and especially during wound healing and tissue repair, as elegantly reviewed by Lendahl et al.^1^.

Fibroblast characteristic elongated spindle or stellate shape presents a multitude of cytoplasmic projections, an oval nucleus, a distinct endoplasmic reticulum and a large Golgi apparatus. When they become activated, they can be defined as “myofibroblasts” (MFB), cells intermediate to fibroblasts and smooth muscle cells with additional distinguished features of nuclear membrane folds and long collections of microfilaments and adhesion molecules connecting them to the surrounding ECM^4,5^.

There are no molecular markers that universally distinguish fibroblasts, however, several cell surface markers are commonly associated with them, such as platelet-derived growth factor receptor-α (PDGFRα) and fibroblast-specific protein 1 (FSP1). Other proteins are more characteristics of activated MFB such as α-smooth muscle actin (αSMA) and fibroblast activation protein (FAP). Nevertheless, none of these markers is universally expressed by all fibroblasts and several are expressed by other cell types (i.e. macrophages), posing a major challenge to unbiased fibroblast identification and isolation. In several studies, fibroblasts are identified and isolated based on negative lineage selection strategies^6,7^.

Despite sharing similar properties and responding to many of the same signalling molecules through common transduction pathways, tissue-specific fibroblasts support the distinctive developmental, physiological and repair needs of their organ of origin. Once fibroblasts populate specific organs, they generate distinct microarchitectures and biophysical and biochemical components of the tissues^3^.

The fibrotic liver stroma is a consequence of sustained liver damage combined with exacerbated ECM accumulation. In this context, activation of hepatic stellate cells (HSCs) triggers profound changes in gene expression in these cells, which play a key role in both initiation and perpetuation of fibrogenesis.

After over a decade of development, single cell and single nucleus RNA-sequencing (sc/sn RNA-seq) have become powerful tools to study transcriptomic profiling with a single cell/nucleus resolution, providing a robust approach to describing cellular identity as well as heterogeneity. In this review, we elaborate HSC and other fibroblast transcriptomic profiles, obtained by sc/sn RNA-seq in development, healthy and disease liver tissue states. We summarise the findings arising from the major sc/sn transcriptomic studies that delineated gene expression profiles of major fibroblast sub-populations in the liver during organogenesis, steady-state and disease, with particular focus on potential functional implications and ECM remodelling activities. A summary of the liver-specific RNA-seq studies discussed in this review is outlined in Table 1, which includes technical details, identified markers and populations, strengths and limitations.

**Table 1:** Summary of the scRNA-seq papers from development to adult in human liver fibroblasts, outlining key strengths and technical challenges of mesenchymal scRNA-seq data^10,23,58,81–85,104–106,109,110,114–117,119,120,144^

	Authors | DOI	Sample source | Data set	Stromal populations	Strengths	Limitations
In development	Popescu et al.| 10.1038/s41586-019-1652-y^10^.	14 foetal livers | Array Express E-MTAB-7407.	**Fibroblast** (ECM1)	27 major cell states identified within the foetal liver, including a fibroblast cell type	Cell types were determined based on 48 genes, with 89% accuracy
	Wang, Yang, Wang et al. | 10.1038/s41422-020-0378-6^144^	9 foetal livers | GSA CRA002443	**Septum transversumal cells** (STCs) (PDGFRA + , NCAM1 + /Pdgfra + , Ncam1 + ) **Hepatic stellate cells** (DCN + , HGF + /Dcn + , Hgf + ), **Mesothelial cells** (PDPN + /Pdpn + )	Weighted gene co-expression network allowed for analysis of both human and mouse liver samples, and comparison of gene regulatory networks	Large differences observed between mouse and human RNA-seq data; further investigation required
	Wesley et al.| 10.1038/s41556-022-00989-7^23^	17foetallivers 16adult livers | Array Express E-MTAB-8210 and E-MTAB-7189	**Stellate cell**(COL1A1, DCN, ACTA2, GFAP, LRAT, HAND2, PDGFRB, RBP1, ECM1)	scRNA-seq analysis revealed the existence of a common progenitor “SEpro” with findings validated in vitro by differentiating human pluripotent stem cells (hPSCSs)	hPSCs within the study do not achieve differentiation into fully mature adult cells in the same manner as in vivo. Following initial differentiation, they continue on an “in vitro” specific path that does not accurately recapitulate liver development
CLD	Ramachandran et al.| 10.1038/s41586-019-1631-3^58^	5 healthy livers 5 cirrhotic livers | GSE136103	**Mesenchyme** (RGS5, PDFRB, LUM, PDGFRA, CCL19)	Unbiased multi-linage approach to characterising the fibrotic niche of liver cirrhosis, identifying novel scar-associated cell markers and pathways relevant to fibrosis such as TNFRSF12A, PDGFR and NOTCH signalling	The 5 sequenced cirrhotic livers are of heterogenous aetiologies, including MASLD, ALD and PBC
	Payen et al.| 10.1016/j.jhepr.2021.100278^81^	2 explanted liver |GSE158723	**Hepatic stellate cell** (COL1A1, COL1A2, CYGB, RGS5, HGF)	2 subpopulations of human HSCs revealed with distinct intralobular localisation, with the GPC3+ HSCs found to express genes relevant to the metabolism of glycosaminoglycans, suggestive of a role in the production and organisation of the liver ECM	Sample size consisted of 2 livers, with 1 explanted sample received from a Crigler-Najjar patient (donor 158) with UMAPs in Supplementary Fig1D showing heterogeneous distribution of the 6 cell populations between the two donors. Unclear if heterogeneity is due to disease, gender, age or other factors. Only 12 HSCs were identified for donor 156 from a total of 9827 cells, representing 0.1% of cells, with limited representation of the liver population
	Andrews et al.| 10.1002/hep4.1854^82^	4 healthy livers | GSE185477	**Mesenchymal** (ADAMTSL2, ACTA2, COL1A1)	7 mesenchymal cell subpopulations identified with snRNA-seq data, and 6 transcriptionally distinct cholangiocyte subpopulations, in comparison to only one population identified through scRNA-seq	Cell dissociation could be responsible for heterogeneity in cell numbers in scRNA-seq; cholangiocytes only made up 0.64% of the cells in the scRNA-seq map despite being an expected 3-5% of the liver cell population
	Fred et al.| 10.1038/s41598-022-16754-7^85^	10 x MASH | Data set not available	**Hepatic stellate cells** (IGFBP7)	Study providing the first transcriptional landscape of MASH from human liver biopsies at a single cell level, and has led to finding of a novel cell type: the subpopulation of macrophages known as Calgranulin expressing, fibrosis-associated macrophages that were found to be upregulated in MASH	Study consisted of 10 MASH livers without comparison with healthy or progressing liver disease
	Filliol et al.| 10.1038/s41586-022-05289-6^83^	2 normal livers 2 MASLD cirrhotic livers 2 adjacent cirrhotic liver tissues 2 HCC liver tissues || GSE174748	Cell clusters identified using PangaloDB and manually clustering based on differentially expressed genes found within their dataset for each cell type	scRNA-seq data revealed the complex role of HSCs in hepatocarcinogenesis with increased HCC risk identified in patients with imbalanced myofibroblastic and HSC populations	Technical artefacts needed to be removed using remove-background function of CellBlender to remove any ambient background RNA or empty droplets present in the raw data files
	He et al.| 10.1530/EC-22-0502^84^	4 healthy livers 2 MASH livers | GSE136103	As stated in the methods section, different cell clusters were grouped by previously defined markers of distinct cells. with some key signature genes identified: **Resting hepatic stellate signature (rHSC)** (RGS5, NDUFA4L2, MYH11, RERGL), **Activated hepatic stellate (aHSC)** (LUM, COL3A1, DPT, PCOLCE), **MASH rHSC signature** (ID3, MALAT1, CRIP1,IGFBP7) **MASH aHSC signature** (IFITM1, MIF, COL1A1, LGALS1)	Transcriptomic changes occurring between activated HSCs and resting HSCs were further elucidated, with MASH-associated transcriptomic signatures identified, including upstream regulators of the functional change occurring in activated HSCs such as SDC1, GRP, SDC4, MUC1	The authors acknowledge that the regulatory roles identified computationally require further experimental validation (e.g. co-culture or knockdown/overexpression experiments)
Cancer	Zhang et al. | 10.1016/j.jhep.2020.05.039^105^	4 treatment-naïve ICC samples, 1 recurrent ICC sample, and 3 adjacent tissues | GSE138709	**Fibroblasts** (ACTA2 and COL1A2)	Defined 6 distinct fibroblast subtypes in human ICC and adjacent tissue, of which vCAFs, mCAFs, iCAFs, apCAFs, and eCAFs	Restrictions in the number of fibroblasts analysed
	Zhu et al.| 10.1038/s41421-023-00529-z^106^	30 HBV-related HCC, 12 frozen HCC tumor tissues, 7 fresh HBV-related and 7 non-HBV related HCC samples | GSE202642	**Fibroblasts** (ACTA2 and COL1A2)	CAF subclusters validated in other HCC scRNA-seq datasets	Relatively low number of CAFs sequenced from HCC tissues
	Zhou et al. | 10.1038/s42003-023-05455-0^110^	3 CCA & 2 HCC | HRA002304, HRA005348	**Fibroblast cells** (TAGLN)	Provides a comprehensive landscape of PLC featuring 3 main tumor types	Sample size could affect the generalisability of findings
	Cheng et al.| 10.1038/s41421-024-00747-z^109^	12 HCC | Data set not available	**Fibroblasts** (COL3A1, DCN, COL1A1)	Established a multimodal approach to investigate the HCC tumour-margin liver axis. Demonstrate that spatially segregated fibroblast subpopulations (CAF-FAP enriched in intratumoral stroma and CAF-C7 enriched in surrounding FRs) critically shape stromal architecture, and that their spatial balance determines clinical outcomes	Validation of the functional roles of RUNX1 and USF2 in vitro was performed on LX-2 cell line which may not fully capture the biological heterogeneity of CAFs within the HCC tumor microenvironment compared to primary CAF cultures
	Lin et al. performed analysis from Ma et al. ^104^| 10.1186/s13099-023-00554-z^117^	13 HCC samples| GSE151530	**CAFs** (DCN, COL3A1, COL1A2)	Found novel CAF-derived gene signatures that play an important role in the development of HCC, for example PTN was a novel mediator of CAFs in HBV cirrhosis-HCC progression	Only 5 HBV-positive patients were derived from the GSE149614 dataset and with this heterogeneous aetiology developing into HCC, further regards into details with viral load, integration patterns and fibrosis stage may need investigation to elucidate these CAF populations’ involvement in HCC progression.
	Chivarina et al., Lin et al., and Tong et al. performed analysis from scRNA-seq data from Ma et al. ^114^| 10.1038/s41388-021-02171-z, 10.1186/s13099-023-00554-z, 10.1186/s12967-024-05613-w^115,116,119^	7 HCC patients and 5 iCCA patients| GSE125449	**CAFs** (COL1A2, FAP, PDPN, DCN, COL3A1, COL6A1)	Focused on intratumoral heterogeneity and detected rare cancer cell subpopulations that would be masked in bulk RNA-seq. This was further explored into the fibroblast population with fibroblast contributions to tumor structure and ECM regulation	Patient-derived CAFs interacting with primary tumor cells could show different regulations compared to exposure for HCC cell lines.
	Yan et al. performed analysis from Lu et al. | 10.1186/s12967-025-06325-5.^120^	10 HCC patients| GSE149614	**Fibroblasts** (ACTA2, COL1A1, COL1A2)	Characterisation of the HCC-TME from different tissue sites of HCC patients showed the heterogeneous nature of CAFs and their fibroblast origins being regionally enriched	CAF origin is inferred from marker expression and spatial localization, and lineage relationships remain to be experimentally validated

## Hepatic fibroblasts and stellate cells in liver development and physiology

### Origin of hepatic fibroblasts and stellate cells during embryonic development

The liver is one of the main organs responsible for metabolism, detoxification, digestion and homeostasis. These critical and vital functions rely on a highly organised and complex architecture that is shaped during its development. The development of the human liver begins early in embryogenesis, around the third post-conception weeks (pcw), from the posterior foregut endoderm, where a region known as the hepatic diverticulum forms and extends into the surrounding septum transversum mesenchyme. Signals from nearby tissues, particularly fibroblast growth factors (FGFs) from the cardiac mesoderm and bone morphogenetic proteins (BMPs) from the septum transversum, are crucial for initiating hepatic specification. By the fifth week of development, the proliferating liver bud invades the mesenchyme, and the early hepatic cords and sinusoidal network that characterize the liver architecture begin to form^8,9^.

During development, between 7 and 17pcw, the foetal liver plays a crucial role in haematopoiesis and haematopoietic stem and progenitor cells (HSPCs)’ expansion^10–12^. The foetal liver niche is a complex and varied microenvironment, with both cellular and extracellular components contributing to HSPCs function and development^13,14^. Liver fibroblasts and HSCs have been identified as potential modulators of haematopoiesis within the foetal liver microenvironment during development, contributing to the haematopoietic stem cell niche^15^. Understanding the emergence and roles of liver fibroblasts and HSCs in modulating foetal liver haematopoiesis is of pivotal importance in our comprehension of the liver niche.

HSCs, located in the space of Disse, have been identified in the human embryo as early as 12pcw^16^. Despite several years of research, the embryonic origin of HSCs remains uncertain as these cells express genes and proteins associated with all three germ layers (mesoderm, ectoderm and endoderm)^17^. The mesodermal precursors of HSCs was identified by lineage tracing studies performed in mice, which found mesothelial and sub-mesothelial cells expressing the Wilm’s tumour suppressor gene (*Wt1*) in the septum transversum mesothelium contribute to generating HSCs and perivascular mesenchymal cells^18^. Evidence also demonstrates that in mice, the mesothelial cells derived from the septum transversum mesenchyme are capable of migrating inwards to generate portal fibroblasts and HSCs surrounding the central vein^19^. Before HSPCs migration into the foetal liver, the mesothelial and sub-mesothelial liver cell populations in mice were found to express desmin (DES), activated leukocyte cell adhesion molecule (ALCAM), p75 neurotrophin receptor (p75NTR), Wt1 and had the potential to give rise to HSCs, expressing DES and p75NTR but not mesothelial markers ALCAM and Wt1, suggesting a shared common precursor to both HSCs and mesothelial cells before differentiation into different lineages^11,19^.

Due to the expression of angiogenic factors by both HSCs and sinusoidal endothelial cells along with their physical proximity in foetal and adult liver, these cells are thought to share a common precursor^20^. This hypothesis was contradicted by observations in zebrafish, where stellate cells were present in the livers of cloche mutants that lacked both sinusoidal endothelial cells and their precursors^21,22^. Furthermore, the presence of HSC progenitors in the early foetal liver has been shown by the expression of PDGFRα and collagen type I (COL1A1), suggesting HSCs progress away from a combined stellate-endothelial progenitor (SEPro) cell lineage^23^. Although the origin of HSC’s is still debated (Table 2), the majority of studies suggests a mesodermal origin, which is supported by studies in mouse, human and avian embryos. As shown by Wang et al. with a thorough analysis of both mouse and human scRNA-seq data, both species suggested the origin of HSCs to be from mesoderm-derived mesenchymal cells. Other studies such as zebrafish, suggest a mesodermal and neural crest origin, which was validated primarily with histological analysis and direct cell lineage tracing and further elucidated with scRNA-seq analysis. Differences in the hypothesised origin of HSCs among species could be due to limitations of the performed lineage tracing experiments rather than species- specific biology, but further research is needed to elucidate this.

**Table 2:** Summary of interspecies differences in fibroblast populations based on single-cell RNA sequencing data^10,19–24,144^

Species	Proposed precursors for HSCs	Markers expressed by HSC’s & HSC precursors	Experimental validation	Conclusions	Author | DOI
Mouse	Mesodermal (mesothelial and sub-mesothelial liver cells)	Mesothelial cells *(Wt1, DES, p75NTR, ALCAM, PDGFRα, COL1A1)*, HSCs *(DES, p75NTR)*	Lineage tracing of Wt1⁺ mesothelial cells strongly suggests a common precursor to HSCs and mesothelial cells before differentiation into different cell lineages.	The identification of a submesothelial cell population and the identification of a mesodermal origin of liver mesenchymal cells. However, further lineage tracing studies needed to confirm mesothelial or submesothelial cells as a precursor for HSCs	Asahina et al. 10.1002/hep.22721^19^
	Mesoderm derived	Hepatic stellate cells *(Zeb2, Tf21, Snai2, Tead2, Nfia, Nfib, Efs1, Zim1)*	scRNA-seq of human and mouse liver cells to identify cell lineage differentiation during hepatogenesis	Diffusion mapping of mouse liver cells found that septum transversumal cells differentiated into HSCs and mesothelial cells, but cell numbers and differentiation pathways varied between human and mouse scRNA-seq liver data	Wang et al. | 10.1038/s41422-020-0378-6^144^
Rat	Common progenitor between sinusoidal endothelial cells and HSCs	VEGF receptor expression (VEGFR1 (Flt-1), VEGFR2 (Flk-1)) and angiogenic responsiveness common between HSCs and sinusoidal endothelial cells	Injury and wound-healing models	Although this focuses predominantly on a wound healing-based model, HSCs and sinusoidal endothelial cell showed functional convergence and coordinated angiogenic behaviour, (which could be suggestive of a shared common origin)	Ankoma-Say et al. | 10.1038/sj.onc.1201912^20^
Zebrafish	Mesodermal and neural crest origin	Expression of *hand2* a neural crest and mesodermal derivative marker	Presence of stellate cells in cloche mutants was found even where sinusoidal endothelial cells (SECs) are not present	Zebrafish liver is not a haematopoietic organ and therefore the difference in function and development must be considered between species. In zebrafish the HSCs are not dependent on SECs or their precursors to originate, however the lack of SECs in the cloche mutant model caused changes in the localisation of HSCs within the developing liver	Yin et al. | 10.1002/HEP.25757^21^
Avian embryos	Mesothelium-derived mesenchymal cells	Mesothelial and endothelial cells (cytokeratin and QH1), HSCs (aSMA)	Direct mesothelial cell labelling of chick embryos to track cell lineage differentiation of mesothelial cells to HSC’s and endothelial cells	Direct cell labelling indicates mesothelial cells invade the submesothelium and become incorporated into the sinusoidal walls with 72 hours, with further analysis of cell specific markers completed with histological analysis	Perez-Pormares et al. | 10.1002/dvdy.10455^22^
Human (Foetal)	Mesodermal/endodermal origin	HSCs *(CD13, CD59, NGFR, Des, aSMA)*, Endothelial (*CD13)*, haematopoietic also express *CD59*	Flow cytometry used to analyse various markers in CD34 + CK7/8+ foetal liver samples to conclude whether this cell population is a common stem cell for haematopoietic and hepatic cells	CD34 + CK7/8+ cells did not express haematopoietic markers but did express hepatic and stellate cell markers which was confirmed with flow cytometry	Suskind et al. | 10.1016/j.jhep.2003.11.007^24^
	Stellate-endothelial progenitor population	Stellate cell *(COL1A1, DCN, ACTA2, GFAP, LRAT, HAND2, PDGFRB, RBP1, ECM1)*	scRNA-seq of human foetal liver tissue to explore hepatic differentiation pathways	Diffusion pseudotime analyses revealed foetal endothelial cells and HSCs could originate from the stellate-endothelial progenitor population with findings further validated using hPSCs	Wesley et al. | 10.1038/s41556-022-00989-7^23^
	n/a	Endothelial cell (*ESAM*, *ECM1*), Fibroblast (*ECM1*), Hepatocyte (*APOA1*)	scRNA-seq of human foetal liver tissue to explore cell differentiation pathways	A fibroblast population is identified by scRNA-seq that clusters closely with other non-immune cell types including endothelial cells and hepatocytes however the suspected fibroblast origin is not expanded upon further	Popescu et al. | 10.1038/s41586-019-1652-y^10^

Despite the several evidence pointing at HSCs being derived from a mesodermal precursor, their expression of markers such as cytokeratin-7/8 and cluster of differentiation 34 (CD34) in the human foetal liver could also indicate a lineage of endodermal origin^24^. Hepatic epithelial cells are also believed to transdifferentiate into HSCs through epithelial-mesenchymal transition (EMT) during liver injury, however, the extent to which EMT contributes to the HSC lineage during development is currently unconfirmed^25,26^. Bone marrow–derived mesenchymal cells and haematopoietic stem cells also appear as possible contributor to the HSCs population. However, the exact cell type of origin and time of differentiation are unclear and controversial and have mainly been observed in injury rather than development^27–29^.

Within the foetal liver, emerging HSCs and fibroblasts show clear distinct profiles^11,30^. For example, HSCs express DES, reelin and B1-integrin, while fibroblasts, do not express reelin, and instead express cluster of differentiation 90 (CD90)^31^. Perivascular fibroblasts of mesodermal origin are also found in the foetal liver, and although their role during haematopoietic development has not been explored in-depth, there is emerging evidence of their influence on shaping the ECM of the foetal liver through the release of proteins such as fibronectins and collagens, which mediate cellular interactions and encourage proliferation of HSPCs^32,33^. HSCs maintain the composition of the ECM in health and disease, by regulating secretion of ECM proteins and ECM-degrading enzymes. During liver development, HSCs contribute to the release of signalling molecules involved in hepatogenesis and haematopoiesis^33^, such as the FMS-like tyrosine kinase 3 ligand (FLT3L) pathway, responsible for influencing foetal liver HSPC production. HSCs also express mRNAs of haematopoietic cytokines such as colony-stimulating factor 1 (CSF1), thrombopoietin (THPO) and erythropoietin (EPO) at various stages of foetal development, and contribute to the establishment of the HSPC niche by releasing many cytokines and growth factors that regulate HSPC expansion including Wnt family growth factors and C-X-C motif chemokine ligand 12 (CXCL12)^11,34,35^. Similarly, studies in mouse and rat models reported the role of HSCs in regulating the HSPC niche in the foetal liver^35–37^.

HSCs and portal fibroblasts also play integral and dynamic roles in orchestrating vascularisation and the differentiation of parenchymal cell types in development^38^. During foetal liver morphogenesis, stromal cells with myoid features, transiently differentiating into MFBs, are especially active within the developing portal tracts^39,40^. These MFBs surround and support nascent bile ducts, hepatic arteries, and portal veins, contributing to the maturation of the portal architecture through ECM deposition and remodelling. As liver development progresses, these cells are replaced by quiescent fibroblasts, highlighting a regulated window of MFB activity during organogenesis. In parallel, HSCs interact closely with both foetal liver endothelial and epithelial cells/hepatoblasts, suggesting a role in maintaining sinusoidal integrity and potentially regulating hepatocyte differentiation via paracrine signalling^41^. Signalling molecules released by HSCs, such as FGFs, have been implicated in the differentiation of hepatoblasts into hepatocytes. Overall, the spatial and temporal specificity of the stromal-epithelial interactions during foetal liver development, underpins proper lobular patterning and vascular organisation, and disruptions in these processes may have implications for congenital liver disorders^8,42^.

### scRNA-seq analysis of HSC and fibroblasts in liver development

The increasing use of single cell omics has allowed for a breadth of information to be obtained regarding the changes in gene expression and cell heterogeneity during foetal liver development. These studies have also provided new evidence on how hepatic cell populations could influence HSPC expansion when the liver is temporarily the primary haematopoietic organ.

**Fig. 1:**
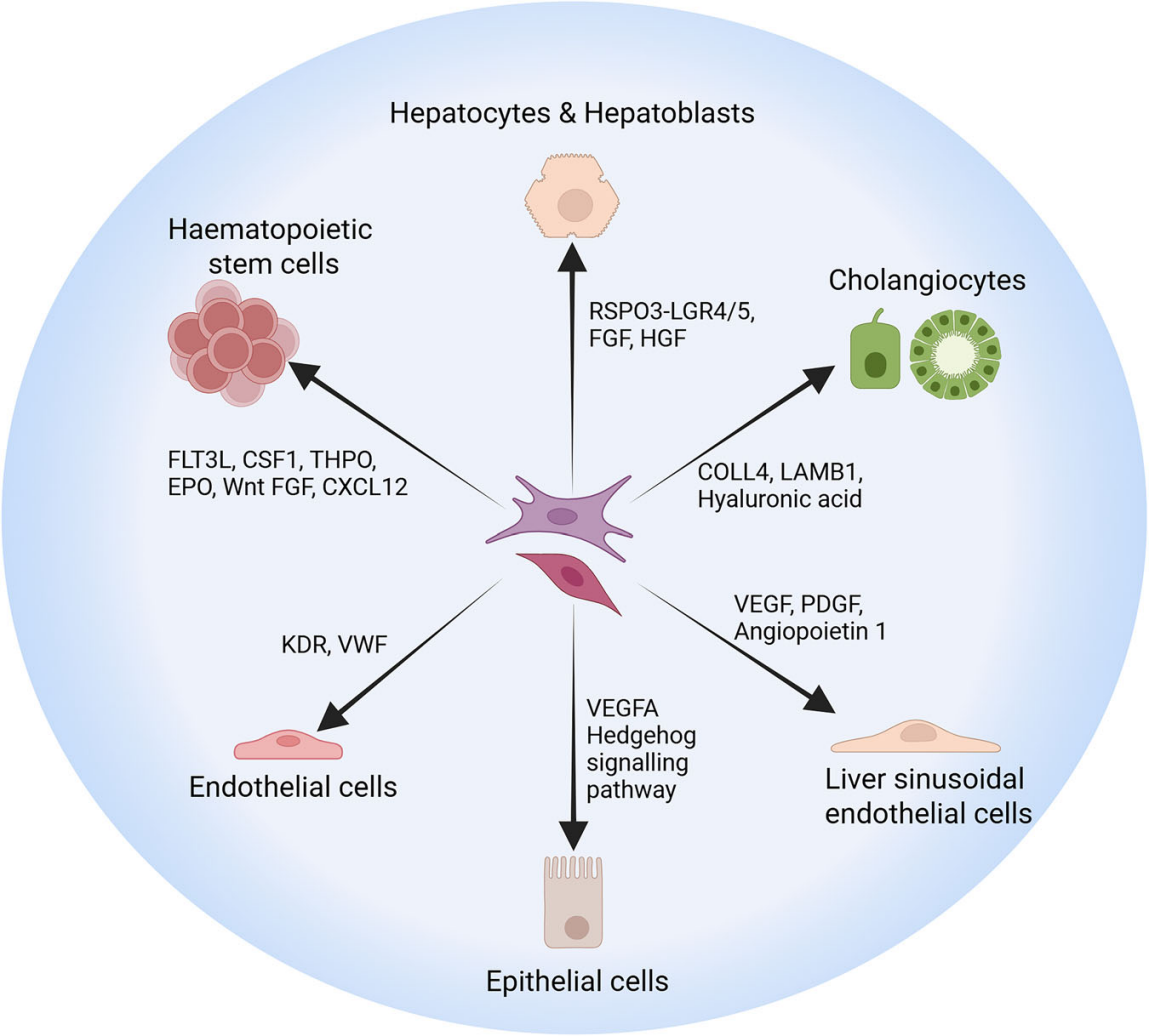
Small-molecule mediators, markers and signalling pathways from fibroblasts influencing the development and differentiation of surrounding cell types. The figure depicts secreted small molecules, including growth factors, cytokines, metabolites, and extracellular matrix components, that act through specific signalling pathways to modulate the fate of surrounding cells. Created with BioRender.

In a scRNA-seq study published by Wesley et al., changes in liver cell types and gene expression throughout development were analysed with a single-cell droplet-based scRNA-seq. HSCs were detected from 5pcw onwards. This study utilised advanced analytical techniques, including Louvain clustering and diffusion pseudotime (DPT) analyses, to trace the developmental trajectory of HSCs. Three distinct populations were identified: one characterized by mesenchymal marker expression, another by endothelial marker expression, and a third that co-expressed markers from both lineages. This suggests the presence of a stellate–endothelial progenitor (SEPro) population that gives rise to both endothelial and stellate cells during liver development. Immunohistochemical validation confirmed the presence of SEpro cells in vivo, co-expressing PDGFRβ and cadherin-5 (CDH5) within the liver vasculature at 6pcw, demonstrating their contribution to early liver formation. In vitro differentiation models with human pluripotent stem cells (hPSCs) were used to further explore stellate cell development. Through scRNA-seq and resulting uniform manifold approximation and projection (UMAP), principal component analysis (PCA), and DPT analyses, the study revealed an overlapping stage during the differentiation of hPSCs into both endothelial and stellate lineages, further suggesting the existence of a shared progenitor capable of transitioning between these cell fates. Functional assays demonstrated that progenitor cells could switch from expressing endothelial markers like *CDH5*, kinase insert domain receptor (*KDR*), and von Willebrand factor (*VWF*) to stellate markers (*PDGFRα, COL1A1*, actin alpha 2 *(ACTA2*), and neural cell adhesion molecule (*NCAM*)), confirming the bipotential nature of these cells^23^. In addition to their developmental origins, stellate cells are also implicated in shaping the hepatic niche and supporting organogenesis. The study identified critical intercellular interactions between stellate cells and other liver cell types, particularly hepatoblasts. For example, the RSPO3–LGR4/5 signalling axis was found to mediate interactions between stellate cells and hepatoblasts at 5–6pcw, suggesting that stellate cells contribute to the self-renewal of hepatoblasts by boosting WNT signaling^23^. This interaction underscores the role of stellate cells in creating a supportive microenvironment for hepatoblast proliferation and differentiation during liver development. The dynamic intercellular interactions involving stellate cells, Kupffer cells, and endothelial cells, may play key roles in ECM organization, haematopoietic development, and immune regulation within the developing liver. These interactions have been further confirmed by other developmental studies such as the study by Yin et al., suggesting that stellate cells are actively involved in coordinating the broader developmental processes of liver tissue formation^15^. Figure 1 provides an outline illustrating how HSCs contribute during development by modulating the fate of surrounding cells. Hepatoblasts and hepatocytes can also influence surrounding stellate cells, establishing a feedback loop that drives functional maturation within the hepatic niche^23^.

Together, these developmental studies indicate that HSCs and fibroblasts do not arise from rigid, terminally fixed lineages, but instead emerge through transient and highly plastic progenitor states that are tightly regulated during organogenesis. In particular, the SEPro population that co-expresses endothelial and mesenchymal markers and gives rise to both lineages during early development, provides a novel conceptual framework for potentially understanding how developmental programmes may be selectively re-engaged in the adult liver during injury, chronic inflammation, or oncogenesis.

In adult liver disease, activated HSCs and fibroblasts upregulate signalling pathways that are prominent during foetal liver development, including wingless-related integration site (WNT), PDGF, transforming growth factor -beta (TGFβ), and NOTCH signalling, which regulate proliferation, migration, and ECM remodelling^15^. In hepatocellular carcinoma (HCC), ECM remodelling activates signalling pathways, such as YAP/TAZ and WNT/TGFβ, which are reminiscent of embryonic developmental pathways and that foster tumour progression and mechanotransduction feedback loop involving fibroblasts and cancer cells. In parallel, endothelial cells undergoing endothelial-to-mesenchymal transition (EndMT) and epithelial cells exhibiting partial EMT acquire mesenchymal-like features, such as increased expression of PDGFRs, collagens, and cytoskeletal genes. While lineage-tracing studies argue against EMT or EndMT as dominant sources of fibrogenic cells in vivo, these processes are increasingly viewed as reversible, adaptive states rather than true fate conversion^43,44^. Notably, experimental studies by Kordes et al. have demonstrated that adult HSCs retain progenitor-like plasticity and can contribute to regenerative responses under specific injury contexts^45^.

Collectively, these observations support a life-cycle model in which stromal cell plasticity is essential during foetal liver development but becomes pathological when embryonic transcriptional programmes are aberrantly reactivated in the adult liver. Framing hepatic fibroblast and HSC heterogeneity within this developmental continuum can provide a unifying perspective that links organogenesis, regeneration, fibrosis, and liver cancer.

### Hepatic fibroblasts and HSCs in the adult liver

In the healthy adult liver, HSCs are present in an inactive or quiescent phenotype^46^. Quiescent HSCs (qHSCs) are non-proliferative HSCs characterised by storing cytoplasmic lipid droplets, which is why HSCs were early named lipocytes. The lipids in qHSCs are mainly vitamin A metabolites or retinoids, contributing to approximately 70%-95% of all retinoids stored in the body. The key HSC vitamin A storage enzyme is lecithin retinol acyltransferase (LRAT). Lrat-deficient mice completely lose HSC lipids and hepatic retinyl ester storage^47^. It is still poorly understood why the loss of lipids in HSCs is considered one of the hallmarks of activation. However, lipid droplet depletion is seen as a consequence of activation rather than a cause^48^. Generally, patients with liver fibrosis or cirrhosis are likely to suffer from symptomatic vitamin A deficiency^47^. Due to the presence of the lipid droplets, qHSCs have similar transcriptional signatures as mature adipocytes^49^, including peroxisome proliferator-activated receptor (PPAR)γ and sterol regulatory-element-binding protein-1c (SREBP1c), which expressions remarkably decreased during fibrogenesis. Clinical data show that pan-PPARγ agonist, pioglitazone, has a positive effect in the reduction of liver fibrosis in metabolic dysfunction-associated steatohepatitis (MASH) patients^50^. In addition to vitamin A storage, qHSCs also have other physiological functions. Due to the predominant role of HSCs in liver fibrogenesis, qHSCs role in contributing to healthy liver ECM homeostasis is easily neglected^51^. In fact, qHSCs located in the Space of Disse produce collagen type I, III, IV and laminin along with a few other ECM proteins. qHSCs also synthesise matrix metalloproteinase (MMP) and tissue inhibitors of metalloproteinase-1 (TIMP1) to keep hepatic ECM homeostasis^52^. They also secrete hepatocyte growth factor (HGF) regulating hepatocyte function. HGF production drops significantly during HSC activation following liver damage, leading to decreased hepatic regeneration in fibrotic livers^53^. Moreover, qHSCs express vascular endothelial growth factor (VEGF), PDGF and angiopoietin 1, regulating liver sinusoidal endothelial cell (LSEC) proliferation^54,55^. Recently, qHSCs were shown having a functional metabolic role in the liver by orchestrating hepatocyte access to lipids via PLVAP, which is highly expressed in HSCs^56^. HSCs also regulate liver architecture and function, influencing hepatocyte zonation and regeneration by producing RSPO3, which modulates WNT signalling^57^.

**Fig. 2:**
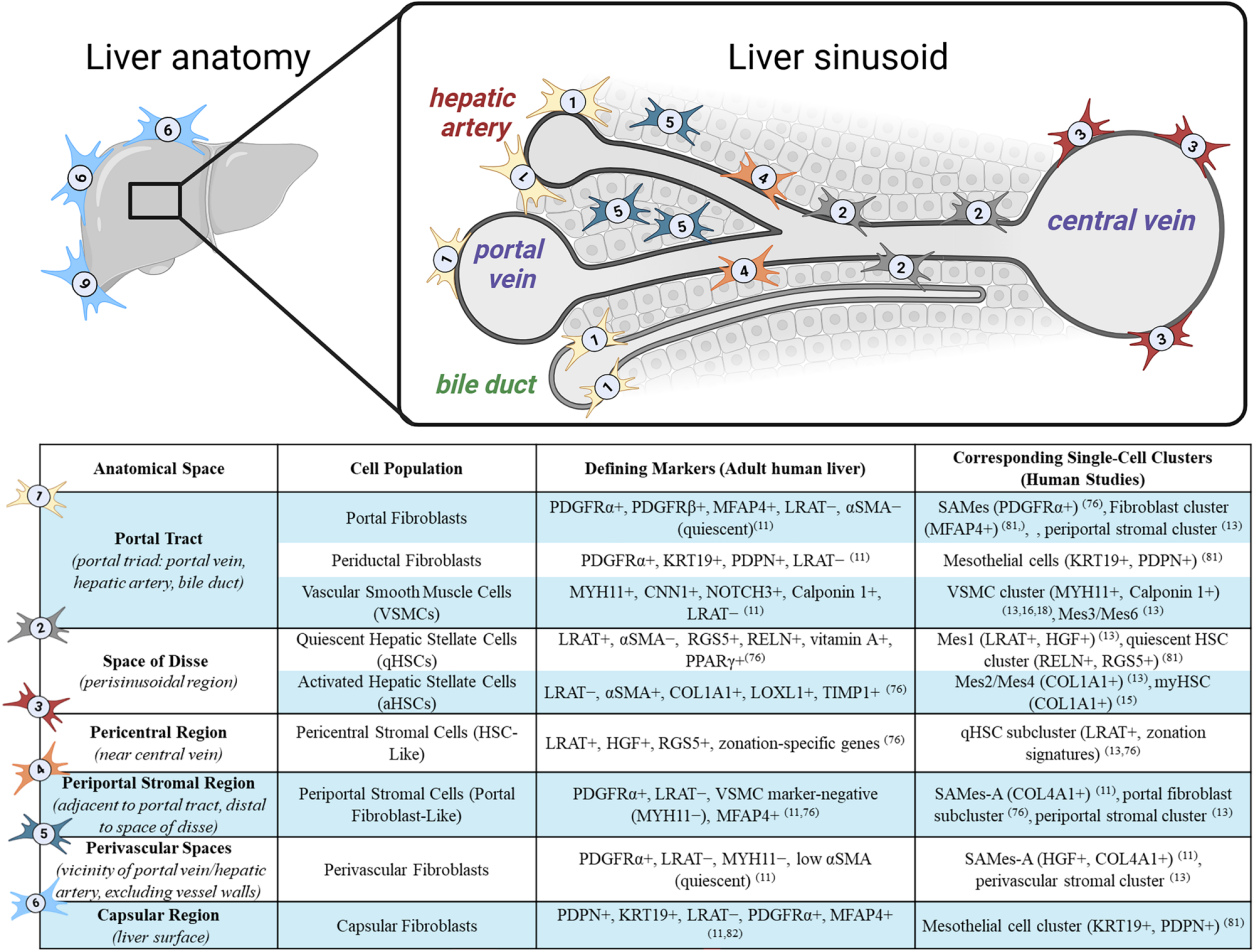
Hepatic mesenchymal cell location and defining markers. Schematic overview showing the distribution of hepatic mesenchymal cell populations, highlighting phenotypic features identified by scRNA-seq data that distinguish these populations^58,59,82,86,145^. Created with BioRender.

In the healthy liver, human hepatic mesenchymal populations have distinct anatomical localisations and phenotypic signatures, as outlined in Fig. 2. Hepatic fibroblasts and their subpopulations (portal, periductal, perivascular, capsular) localise to non-perisinusoidal regions, characterized by LRAT negativity and expressing markers like PDGFRα, MFAP4, and PDPN^58^. HSCs reside only in the Space of Disse: quiescent HSCs express LRAT, while activated HSCs lose LRAT and upregulate αSMA^59^. Pericentral and periportal stromal cells show HSC-like and portal fibroblast-like phenotypes, respectively. Portal fibroblasts are also found in a quiescent state, surrounding the portal vein and contributing to the maintenance of the integrity of the portal tract, and around the bile ducts. Unlike qHSCs, portal fibroblasts are negative for LRAT, as they have no role in storing retinol droplets. Similarly to qHSCs, portal fibroblasts are highly metabolically active^60^. They are key regulators of epithelial patterning, providing positional information, maintaining epithelial cell phenotype and migration^61^, regulating cholangiocyte proliferation via hyaluronic acid production^62^, and participate in the putative peribiliary stem cell niche^63^. Vascular smooth muscles cells (VSMCs) are identifiable by MYH11 and CNN1^58^. Single-cell clusters validate these differences, confirming anatomical niche-determined cell identity, distinguishable via markers and cluster associations^58,59^.

### Activation of HSCs and transdifferentiation into myofibroblasts in chronic liver disease

Chronic liver disease (CLD) is defined as a progressive deterioration of liver function for more than six months, and it causes approximately 2 million deaths globally every year^64^. CLD is the result of a continuous process of inflammation, destruction, and regeneration of liver parenchyma, which leads to fibrosis and cirrhosis^65^. Nowadays, the most common triggers of CLD are excessive consumption of alcohol, hepatic fat accumulation and hepatitis B and C virus infection, causing ALD, metabolic dysfunction-associated steatotic liver disease (MASLD), and hepatitis B and C (HBV and HCV), respectively^66^.

When liver injury happens, HSCs are activated through an initiation phase, followed by perpetuation. When the liver injury is resolved, activated HSCs (aHSCs) undergo a third phase, which is either resolution by senescence, apoptosis or reversion to quiescent stages^66,67^. The first phase, initiation of HSCs activation, is also called pre-inflammatory stage. HSC migration is normally the initial step in response to liver injury, guided by fibronectin (FN1) and various growth factors, such as TGFβ, epidermal growth factor (EGF) and PDGF^67,68^. A number of immune cells also regulate HSC response and activation, in particular T cells^69,70^ and macrophages^71,72^. Changes to ECM composition and stiffness orchestrated by aHSCs can further impact other qHSC response to liver injury, establishing a feed-forward loop^51,73^. Mechano-responsiveness in hepatic and cancer fibroblast (see section below) subpopulations facilitates a self-reinforcing feedback loop where increasing ECM stiffness and topographical signals drive the transition from quiescence to activated, heterogeneous states that further remodel the fibrotic and cancerous microenvironment. HSC initiation promotes transcriptional changes to alter the microenvironment at the injured region, therefore promoting HSC transition to the second phase, perpetuation. Proliferation then occurs, which leads to an increase in the absolute number of HSCs with a profibrogenic phenotype. TIMPs released by HSCs can also promote proliferation and maintain HSCs survival^74^. αSMA expression with stress-fibre-like appearance is another feature of the transition from HSCs to MFBs through TGFβ pathway. aHSCs tend to migrate through HSC cytoskeletal remodelling towards the injured region enriched with PDGF, EGF, TGFβ, chemokine (C-C motif) ligand 2 (CCL2) and other growth factors and inflammatory cytokines^75–77^.

ECM production is regarded as a hallmark of HSC transition to MFB, in particular COL1A1 production that replaces COL4A1 as one of the basement membrane components in CLD^78^. As the main source of MFB in CLD fibrosis, HSCs contribute approximately to 82-96% of all MFB, as evidenced by a study using a murine model of toxic, cholestatic and fatty liver disease^79^. However, LRAT-negative portal fibroblast-like cells were found to be the most important source of MFB around the bile duct than HSCs in cholestatic liver injury^79,80^.

The same dramatic phenotypic activation and pathophysiological mechanisms described for activating HSCs are less studied in fibroblasts, or it remains unclear if these stages are shared by HSCs and hepatic fibroblasts.

### Sc/snRNA-seq analysis of HSC and fibroblasts in homeostasis and CLD insurgence

One of the first studies illustrating fibroblast transcription profiles by scRNA-seq was conducted by Ramachandran et al. in 2019^58^. The authors classified four subpopulations of fibroblasts in both healthy and cirrhotic livers, which were VSMCs (myosin heavy chain 11 (*MYH11*), HSCs (regulator of g-protein signalling 5 (*RGS5*)), scar-associated mesenchymal cells (SAMes) *(PDGFRα)* and mesothelial cells (keratin 19 (*KRT19*), podoplanin (*PDPN*)). During activation and transdifferentiation to SAMes, HSCs exhibited a reduced expression of *RGS5*, insulin-like growth factor binding protein 5 (*IGFBP5*), GTP-binding protein overexpressed in skeletal muscle (*GEM*), with an upregulation of lysyl oxidase like 1 (*LOXL1*)*, COL1A1, COL1A2* and collagen type III (*COL3A1*). The population of SAMes were further subclustered into SAMes-A and -B. Although SAMes-A population was identified in both healthy and cirrhotic liver with expression of *HGF* and collagen type IV (*COL4A1, COL4A2*), this fraction was significantly higher in the cirrhotic liver as opposed to the healthy one (~ 6-fold increase). On the other hand, SAMes-B population was only reported in cirrhotic livers, with expression of fibrillar collagens (*COL1A1, COL1A2* and *COL3A1*).

In 2021, Payen et al.^81^ also described the HSC heterogeneity in the healthy human liver. After human liver dissociation, approximately 1% of the total number of cells analysed (25,325) were annotated as HSCs and separated into 2 subclusters, HSC1 and HSC2. Both HSC subpopulations expressed typical HSC markers, including *RGS5*, *LRAT*, and decorin (*DCN*). Although several activation markers were detected in HSCs from healthy livers, such as *ACTA2*, *COL1A1*, lysyl oxidase (*LOX*), and *TIMP1*, these markers were expressed by both HSC1 and HSC2 clusters. GO analysis showed that HSC1 expressed GAGs metabolism-related genes lumican (*LUM*), glypican 3 (*GPC3*), versican (*VCAN*) and cluster of differentiation 44 (*CD44*) and elastic fibre constitutes related genes fibulin 1 (*FBLN1*), fibulin 5 (*FBLN5*) and elastin (*ELN*), while HSC2 population expressed antigen presentation genes human leukocyte antigen – DRβ1 (*HLA-DRβ1*), human leukocyte antigen – DRα (*HLA-DRα*) and cluster of differentiation 74 (*CD74*). These results indicated that HSC heterogeneity in the healthy liver was mainly driven by physiological processes other than activation status.

In the same year, Andrews et al.^82^ performed both scRNA-seq and snRNA-seq to comprehensively understand healthy human liver cell transcriptomic profiling, illustrating for the first time the heterogeneity of all mesenchymal cell clusters in the liver with various activation degrees at single-cell level. Seven clusters were identified: Mes1 to Mes7. Mes3 and Mes6 were described as VSMC with expression of *ACTA2* and myosin light chain 9 (*MYL9*). Mes6 exhibited a more activated VSMC phenotype with collagen formation, ECM and integrin signalling genes (*COL1A1*, *COL3A1*, secreted protein acidic and rich in cysteine (*SPARC*)), while Mes3 had a higher expression of angiogenesis and epithelial proliferation genes (vascular endothelial growth factor A (*VEGFA*)). Mes1 was classified as qHSC with expression of *LRAT*, *HGF* as well as neuronal pathway-related genes. During the process of activation, Mes5 displayed a pre-activated HSC phenotype with expression of qHSC markers, lipid transport and organic acid metabolism, inflammation-associated genes and antigen presentation. Both Mes2 and Mes4 were reported to express myofibroblast-associated genes (*ACTA2*, collectin subfamily member 11 (*COLEC11*)). However, Mes2 expressed higher level of cytokines and growth factor and their receptors (*TGFβ1* and *TGFβR2*) as well as more lipid metabolism genes (aldehyde oxidase 1 (*AOX1*), phosphodiesterase 3D (*PDE3D*)) than Mes4, while Mes4 expressed more collagen production and matrix remodelling genes (*COL1A1*, *FN1* and *SPARC*) as well as senescence and inflammation related genes (interleukin 32 (*IL32*), *CCL2*), which indicated Mes4 were more activated than Mes2. Mes7 was identified as doublet by expression of retinol storage genes and lymphocyte-associated transcripts.

In another study by Filliol et al., which focused on the classification of HSCs in healthy human and MASH-related cirrhotic livers, two main populations were identified: cytokine-producing HSCs (cyHSC) and myofibroblast HSCs (myHSC). CyHSCs expressed *LRAT*, *HGF*, reelin (*RELN*) and *RGS5*, exhibiting a quiescent phenotype, while myHSC expressed collagens and ECM protein-related genes, such as *COL1A1* and *ELN*^83^.

He et al. also provided the transcriptional profiling of HSCs in MASH-related fibrosis by regrouping and analysing the GSE136103 dataset by Ramachandran et al. Interestingly, although they identified several VSMC-specific markers (*MYH11*, calponin 1 (*CNN1*) and neurogenic locus notch homolog protein 3 (*NOTCH3*)) from resting HSC subclusters, VSMC were not separated from the HSC pool, and the cluster was not named more inclusively. The other HSC subcluster identified was the aHSCs, which were reported expressing collagen production and ECM remodelling genes, such as *LUM*, *COL3A1*, and *LOXL1*. When compared to aHSC from healthy liver, aHSC from MASH patients expressed a higher level of collagen type I (*COL1A1* and *COL1A2*), while *COL3A1* and collagen type VI (*COL6A3*) appeared in aHSC signature from both healthy and MASH livers. Other ECM-related genes, including *FN1*, *TIMP1*, and *SPARC*, were only identified as MASH-aHSC transcriptional signature^84^.

Fred et al. broadly characterised HSCs from MASH patients into three subpopulations: quiescent, intermediate, and myofibroblast. Within the activated HSC population of MASH patients, two distinct populations were identified, being either *ACTA2*^*+*^ or *RBP1*^*+*^. *RBP1*^*+*^ activated HSCs were highly associated with ECM formation and fibrosis formation in MASH due to the enrichment of collagens and the two matrix remodelling genes *TIMP1* and *TIMP3*^85^.

Interestingly, single-cell meta-analyses of some of these studies were conducted to increase power, define conserved cell subtypes across donors and disease aetiologies, find potential biomarkers or therapeutic targets, and enable cross-study comparisons. Merens and colleagues integrated three human scRNA-seq studies mentioned above to construct a single-cell atlas of HSCs, which identified a strongly conserved HSC activation process with three major HSC states/subtypes: quiescent, initiatory, and myofibroblasts. Quiescent HSCs expressed canonical quiescent markers (*RELN, ECM1, RGS5*) and G-protein-coupled receptor genes (*VIPR1, CALCRL, EDNRB, AGTR1, PTH1R*) and other genes including *FCN1, COLEC10, COLEC11*. Initiatory HSCs expressed chemo-attractant cytokine *CCL2, THBS1* (YAP target), and *TIMP1*. These cells represent an intermediate activation step between quiescent and fully myofibroblastic, which were characterised by genes such as *S100A6, WT1, C3*, and classical fibrogenic / collagen genes (e.g., *COL1A1*, etc.). The meta-analysis confirmed the loss of quiescent/zonation signatures and gain of proliferative, migratory, ECM-producing programs as the hallmark of activation, across aetiologies and studies. Importantly, zonation signatures (portal vs central) appeared in the quiescent state and were lost in later states^59^.

Similarly, Li et al.^79^ generated the multi-modal integrative liver expression atlas GepLiver of human (and mouse) tissues, single cells and cell lines. The atlas annotated four main liver mesenchymal cell types: VSMCs, defined by marker gene *MYH11*, HSCs *(RGS5-*positive), mesothelial cells, defined by the expression of *KRT19*, and scar-associated mesenchymal cells (reported enriched in liver cirrhosis), expressing *COL1A1*^86^. The scar-associated mesenchymal cells seemed to correspond to the myoHSC delineated by Merens et al.^59^.

These examples of meta-analyses confirmed that the activation and gene programs appear share across injury types, suggesting that early activation may be an attractive target for a broadly applicable antifibrotic strategy. Meta-analysis articles highlighted the need to start integrating individual studies to harmonise mesenchymal cell definitions, although they do not deeply dwell on functional phenotypes of each mesenchymal subtype beyond abundance dynamics and marker annotation. Integrative analyses represent important approaches to provide references of subtype identity, identification of biomarkers, and relative changes across phenotypes.

### Hepatic fibroblasts in cancer

Upon chronic liver damage, myofibroblasts undergo a series of transitions, becoming more metabolically active and leading to a fibroblast population resembling cancer-associated fibroblasts (CAFs)^87^. Compared to normal fibroblasts, CAFs exhibit distinct morphological characteristics, which is a common form of identification between the two cell types. Normal fibroblasts typically have a uniform spindle shape with minimal cytoplasmic extensions, small oval-shaped nuclei and they have a normal distribution of cellular organelles. CAFs typically have a spindle-shaped appearance with multiple cytoplasmic extensions, larger indented nuclei, abundant ribosomes, an expanded rough endoplasmic reticulum, and a more developed Golgi apparatus, indicative of abundant protein synthesis^88^. CAFs are typically defined by the following criteria: (1) they are mesenchymal cells derived from tumours; (2) they are negative for epithelial, endothelial, and leukocyte markers; (3) they possess an elongated shape; and (4) they lack the cancer cell mutation genes^89,90^.

### CAF functions

CAFs are the predominant cell type in the tumour microenvironment (TME), typically viewed as tumour-promoting, however, in some cases they have been identified to exhibit tumour-inhibiting properties. For instance, αSMA, a common CAF-marker has a dual role dependent on the cancer origin. In liver cancer, αSMA^+^ CAFs promote tumour progression by altering the TME, leading to poor prognosis^91^. Conversely, in pancreatic ductal adenocarcinoma (PDAC), the loss of αSMA^+^ CAFs increases the number of CD4^+^FOXP3^+^ T-reg cells, leading to an increase tumour mass, highlighting their tumour-suppressive role^92^. Several studies have continued to show the function of CAFs in PDACs progression and their different roles in therapy response by being tumour-promoting or tumour-restrictive; however, this has not been further elucidated in liver cancer^93,94^. In the hepatic TME, CAFs hinder anti-tumour immunity by secreting cytokines, chemokines and growth factors such as interleukin 6 (IL6), CXCL12, and TGFβ that directly or indirectly support cancer cells and induce ECM remodelling to enhance tumour cell proliferation and survival, creating an immunosuppressive network^95–97^. The balance being tumour-promoting or tumour-restrictive functions can shift over time, and it is influenced by tumour stages, microenvironment changes and interactions with other cells within the TME, making CAFs a critical player in liver cancer progression^87^. The lack of precision around fibroblast-specific markers poses a challenge when considering the origin of CAFs. Foster et al. suggested that CAF subpopulations across solid tumour types (breast, colon, lung, skin and pancreatic) fall into three broad categories: steady-state like, mechanoresponsive and immunomodulatory, which are governed by changes in chromatin accessibility and are spatially distinct^98,99^. Mechanical responsiveness contributes to CAF heterogeneity, where distinct subpopulations (e.g., myofibroblastic CAFs) respond to physical signals by upregulating αSMA and collagen synthesis to drive matrix remodelling^100^. Mechanotransduction pathways, specifically the Hippo-YAP/TAZ signalling axis, act as critical mediators; high matrix stiffness inhibits upstream Hippo kinases, allowing YAP/TAZ to translocate to the nucleus and activate transcriptional programs for cell proliferation and ECM production^101,102^. Beyond stiffness, the topography and 3D architecture of the ECM, including fibre alignment and mechanical confinement, provide contact guidance for fibroblasts^103^.

Single-cell transcriptomic data from Foster et al. and Ying et al. have revealed complex, heterogeneous subtypes of CAFs that exhibit conserved characteristics across different primary liver cancers dependant on their precursors. As a highly heterogeneous population of fibroblasts, they seem to originate from various sources of tissue-resident fibroblasts and stellate cells which have been activated upon exposure to tumour cell production of factors including TGFβ, PDGF and FGF, instigating a mesenchymal-mesenchymal transition^98,99^.

### CAF RNA-sequencing analysis in primary liver cancer

In primary liver cancers, such as intrahepatic cholangiocarcinoma (ICC) and hepatocellular carcinoma (HCC), CAFs exhibit distinct characteristics driven by their specific microenvironments. HCC is derived from the malignant transformation of hepatocytes and CCA is thought to originate from the malignant transformation of cholangiocytes. CCA is generally classified as intrahepatic and extrahepatic. Among these, intrahepatic cholangiocarcinoma (iCCA) together with HCC is considered one of the most common primary liver cancers. Both forms of cancer develop from similar aetiologies and commonly occur in people with CLD, notably with a background of cirrhosis^99^. Understanding the unique subtypes of CAFs and their interactions with tumour and immune cells is crucial for elucidating their roles in cancer biology and developing targeted therapies.

Zhang et al. identified five CAF subpopulations and performed gene ontology analysis to decipher the CAF-cellular diversity within iCCA. Vascular CAFs (vCAFs), the most abundant fibroblast subpopulation (57.6%) enriched for microvasculature genes (*IL6*, CC-motif chemokine ligand 8 (*CCL8*)) were linked to muscle contraction, hypoxia response, and mesenchymal proliferation. Matrix CAFs (mCAFs), marked by ECM-related genes, were associated with collagen organisation. Inflammatory CAFs (iCAFs), expressing *FBLN1*, insulin-like growth factor-I (*IGFI*), C-X-C motif chemokine ligand 1 (*CXCL1*), insulin-like growth factor binding protein 6 (*IGFBP6*), secretory leukocyte protease inhibitor (*SLPI*), serum amyloid A1 (*SAA1*), and complement genes (*C3, C7*) were associated with inflammatory regulation and complement activation. Antigen-presenting CAFs (apCAFs), characterised by antigen presentation were involved in leukocyte adhesion and MHC-II pathways; and EMT-like CAFs, expressing *KRT19*, keratin 8 (*KRT8*), *SAA1*, showed EMT-like signatures^104^. These subpopulations have also been identified in other studies, further validating their presence and classification^105^.

In HCC, a study by Zhu et al. described five common CAF subtypes: vCAFs, mCAFs, lipid processing mCAFs (lpmCAFs also called *CD36* + CAFs), lipid-processing CAFs (lpCAFs), and apCAFs, with a particular emphasis on the newly identified *CD36*^*+*^ CAF subset. They derived the pseudotime cell trajectory of the various CAF subtypes and observed dynamic transitions from the progenitor state leading to the distinct terminal effect state. CAFs are positioned in the progenitor state, indicating their role as precursors in the differentiation process. From this state, they give rise to intermediate lpmCAFs, which then differentiate into various terminal subtypes, such as vCAFs, lpCAFs, and apCAFs. As CAFs progress along the trajectory, specific gene expressions change significantly. Notably, mCAFs show decreased expression of ECM genes such as *LUM*, *DCN*, *VCAN*, and periostin (*POSTN*), while genes associated with terminal states, including apolipoprotein C1 **(***APOC1*), cluster of differentiation 74 (*CD74*), and *HLA-DRα*, were upregulated. Transcription factors linked to lipid metabolism and antigen presentation, such as CCAAT enhancer binding protein alpha (*CEBPA*), V-maf musculoaponeurotic fibrosarcoma oncogene homolog B (*MAFB*), and Ikaros zinc finger protein 1 (*IKZF1*), were gradually upregulated during differentiation. In contrast, factors like CCAAT/enhancer-binding protein beta (*CEBPB*), nuclear factor IC (*NFIC*), Twist-related protein 1 (*TWIST1*), and CAMP-responsive element binding protein 3 like 1 (*CREB3L1*) were downregulated, suggesting their involvement in regulating the transition and transdifferentiation of CAFs^106^.

Generally, in scRNA-seq CAF-associated research, including studies on HCC and CCA, the nomenclature used to identify and characterise CAFs often overlap when referring to similar cellular features and functions. Yet varied nomenclature and marker panels are still employed to further characterise additional subpopulations^107^.

Following scRNA-seq analysis by various groups, functional analysis studies have been undertaken to decipher the roles of CAFs between the two different primary cancers, based on the broader characterisation of their subpopulations. Functional analyses revealed that CAFs in iCCA and HCC play distinct roles within the TME. CAFs from iCCA were enriched in functions related to morphogenesis and matrix remodelling functions, whereas HCC-derived CAFs showed features related to immune activation and metabolism. This finding aligns with a key pathological hallmark of iCCA: a pronounced desmoplastic reaction, characterised by a dense, fibro-collagenous stroma where CAFs are the most abundant mesenchymal cells^108^. Additionally, the higher proportion of CAFs in iCCA indicates their potential involvement in promoting a more fibrotic environment that supports tumour growth and metastasis.

Cheng et al. identified two key fibroblast subpopulations in HCC: CAF-FAP, enriched in the intratumor stroma, and CAF-C7, predominantly situated in the surrounding fibrotic ring. CAF-FAP showed high expression of *FAP*, *POSTN*, thrombospondin-2 (*THBS2*), and NADPH oxidase 4 (*NOX4*) and functionally resembled iCAFs, with upregulated chemokine signalling and pro-inflammatory genes (*CCL2*, *CXCL12*, growth/differentiation factor 15 (*GDF15*), LIF-receptor subunit alpha (*LIFR*)) at the tumour core. In contrast, CAF-C7, marked by elevated *C7* and *PDGFRα*, mirrored myofibroblast CAFs (myCAFs) and displayed chemokine signalling alongside pro-fibrotic genes (Tumor necrosis factor receptor superfamily member 12 A (*TNFRSF12A*), *TGFβR1*, *TGFβR2*) at the fibrotic ring. Additionally, HSCs shared features with apCAFs. Notably, upon examining the human HCC TMAs, the spatial balance between CAF-FAP and CAF-C7 was linked to clinical outcomes with higher CAF-FAP at tumour core or lower CAF-C7 at the tumour margin exhibiting worse survival^109^. Pseudotemporal ordering indicated that iCCA-derived and HCC-derived CAFs were located at opposite ends of the trajectory, further confirming their distinct roles in the tumour microenvironment^110^.

### Origin of the heterogeneous populations of CAFs

**Fig. 3:**
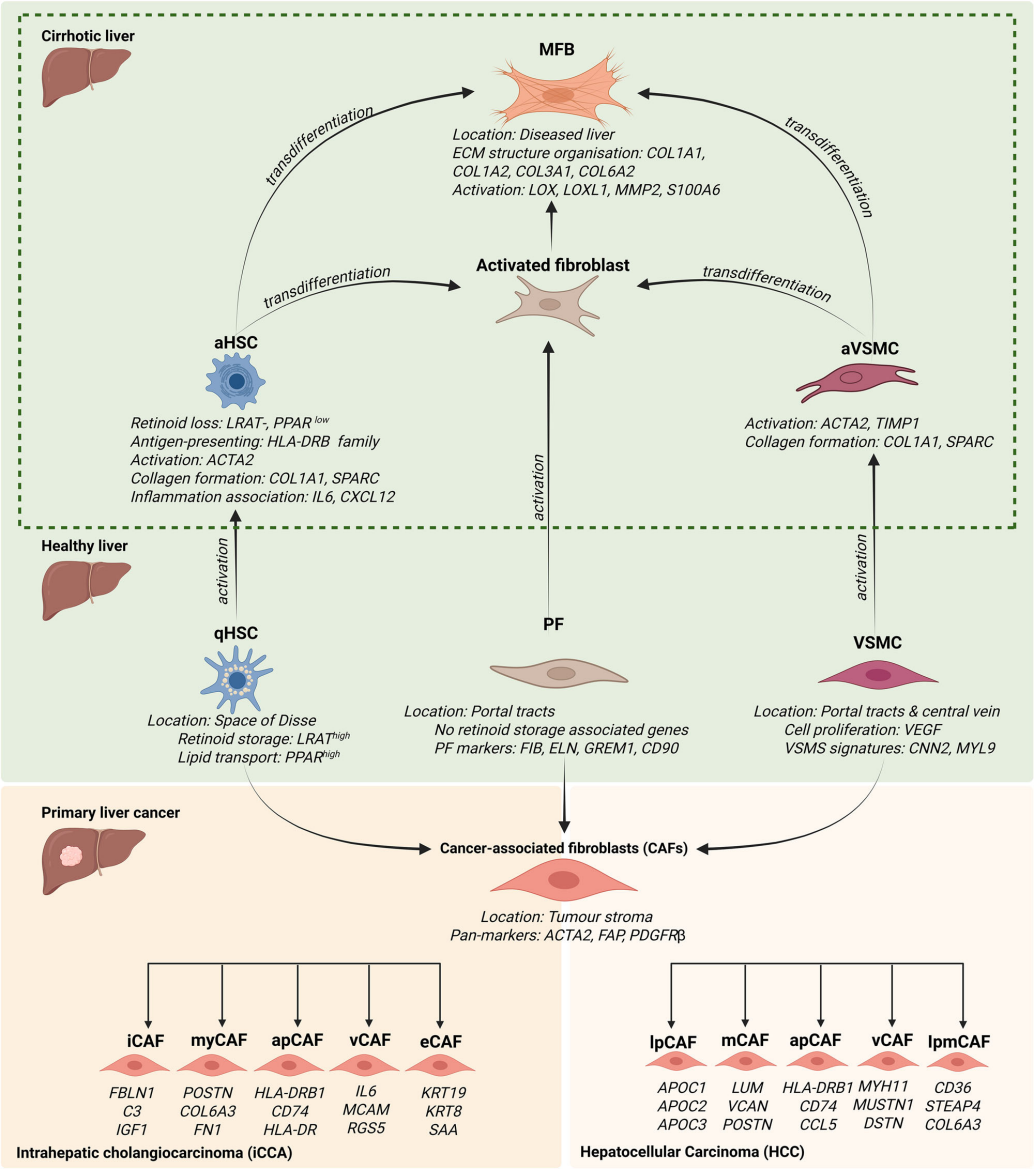
Overview of the origins and identifiable markers of adult hepatic fibroblasts from homeostasis to activation in disease. Hepatic fibroblast and stellate cell dynamics in the progression to cirrhosis and liver cancer. Hepatic stellate cells (HSCs) are the primary source of myofibroblasts and cancer-associated fibroblasts (CAFs) in cirrhosis and cancer, respectively. Portal fibroblasts (PF) and vascular smooth muscle cells (VSMC) are less abundant cells within the liver, but both can influence disease progression upon activation or transdifferentiation. The dotted line indicates fibroblast populations shared between healthy and cirrhotic livers, with their abundance varying according to disease state. CAFs represent a highly heterogeneous population within the tumour liver microenvironment, and based on single-cell analysis, they have been divided into five broad categories to distinguish them between function in both iCCA and HCC. iCAF (inflammatory), myCAF (myofibroblast), apCAF (antigen-presenting), vCAF (vascular), eCAF (EMT-like), mCAF (matrix producing), lipCAF (lipid-processing), lIpmCAF (lipid-processing). Created with BioRender.

Multiple cell types including HSCs, PFs, mesenchymal stem cell–like cells, and bone marrow–derived cells, have been proposed as contributors to the hepatic myofibroblast pool. However, the cellular origins of CAFs in HCC and CCA remain incompletely defined and appear highly heterogeneous. Research using gene tracking in mouse models has revealed that CAFs primarily originate from HSCs in both HCC^111^ and ICC^112^, followed by other mesenchymal cells such as portal fibroblasts^58,113^. Figure 3 provides an overview of the origins of adult hepatic fibroblasts, underpinning their progression from homeostasis to activation in disease and cancer. Nonetheless, a comprehensive comparison of fibroblast phenotypes derived from distinct sources in liver cancer remains absent.

Chiavarina and colleagues identified three subtypes of HCC-CAFs, from scRNA-seq analysis of previously published dataset GSE125449, each linked to distinct cellular origins: hepatic stellate cells (CAF_HSC), vascular smooth muscle cells (CAF_VSMC), and portal fibroblasts (CAF_Port). Among these, the CAF_Port subtype, though representing a minor population, uniquely expressed prolargin (*PRELP*) mRNA. This was further supported by immunohistochemical staining, which showed PRELP protein specifically localized in the portal regions of healthy liver tissue, with no expression in liver sinusoids. Functionally, *PRELP*, a small leucine-rich proteoglycan, demonstrated tumour-suppressive properties in HCC, primarily by inhibiting angiogenesis^114,115^. Recent research has shed light on the role of secreted phosphoprotein 1 (*SPP1*) in this process, highlighting how tumour cells exploit cellular interactions to manipulate their microenvironment. Using an HCC scRNA-seq database, it was found that tumour cells expressing high levels of *SPP1* were correlated with increased fibroblast presence. Spatial transcriptomic data revealed that the *SPP1* secreted by HCC was located near HSCs, where it binds to the CD44 receptor expressed by HSCs. This interaction activates the PI3K/AKT signalling pathway, leading to the expression of fibrogenic markers such as *αSMA, FAP*, and *FSP* promoting the differentiation of HSCs into CAFs. This SPP1-CD44 interaction underscores a key mechanism by which tumour cells manipulate the TME to support tumour growth. These findings suggest that targeting the SPP1-CD44-PI3K/Akt pathway could be a potential therapeutic strategy to inhibit CAF activation, reduce fibrosis, and slow tumour progression in HCC^116^.

Pseudotime analysis revealed that fibroblasts and endothelial cells appear earlier than B cells and CD8^+^ T cells in the TME, suggesting their critical role in the early stages of HCC development and tumour progression. Single-cell transcriptomic data from the GSE151530 dataset were utilised to classify fibroblasts into three distinct subclasses (CAF0-2) from 57 genes. Through integrated data analyses, CAF2 (*PTN*^*+*^*, SDC1*^*+*^*, NCL*^*+*^) demonstrated strong interactions with immune cells, particularly B cells and CD8^+^ T cells, suggesting that this subclass of CAFs contributes to the remodelling of the immune microenvironment. The *PTN* identified in this study may serve as a CAF-specific biomarker and a key molecular mediator in the progression of HBV-associated fibrosis, cirrhosis and HCC^104,117^. Additionally, Park et al. showed that *PTN* is expressed in HSCs, indicating that PTN-expressing HSCs may transform into CAF-PTN and contribute to modulating immune responses within the TME^118^. Recently, Yan et al. performed analysis based on the GSE149614 dataset and showed that during the progression from liver fibrosis to HCC, *YAP1*^+^ CAFs, which were most abundant in tumour tissues, displayed significantly elevated expression of *COL1A1* and *COL3A1*, and further upregulated matrix rigidity-associated genes such as *LOX*, collagen type XII (*COL12A1*), *COL4A1*, collagen type V (*COL5A3*), *COL6A3*, and collagen type IX (*COL9A3*), thereby promoting liver cancer progression^119,120^.

Similarly, Affo et al. demonstrated that HSC-derived CAFs represent the principal tumour-interacting fibroblast population in iCCA. To further understand the origin of CAFs, the group investigated PDAC-derived CAFs (which have been reported to be tumour-restrictive). Although pancreatic stellate-cell-CAFs weakly expressed canonical markers such as *Lrat*, *Des*, or *Rgs5*, they shared a global stellate cell gene signature with HSC-CAFs, supporting a common stellate lineage. Notably, this suggests conserved CAF ontogeny across the fibrotic organs that harbour stellate cells^112^. More recently, Yin and colleagues used Visium HD spatial transcriptomics to identify endothelial cells co-expressing CAF marker genes and inferred developmental trajectories linking endothelial cells to CAFs. These findings suggest that EMT, potentially associated with CTGF signalling, may represent an additional cellular origin of CAFs in HCC^121^.

## Discussion

**Fig. 4:**
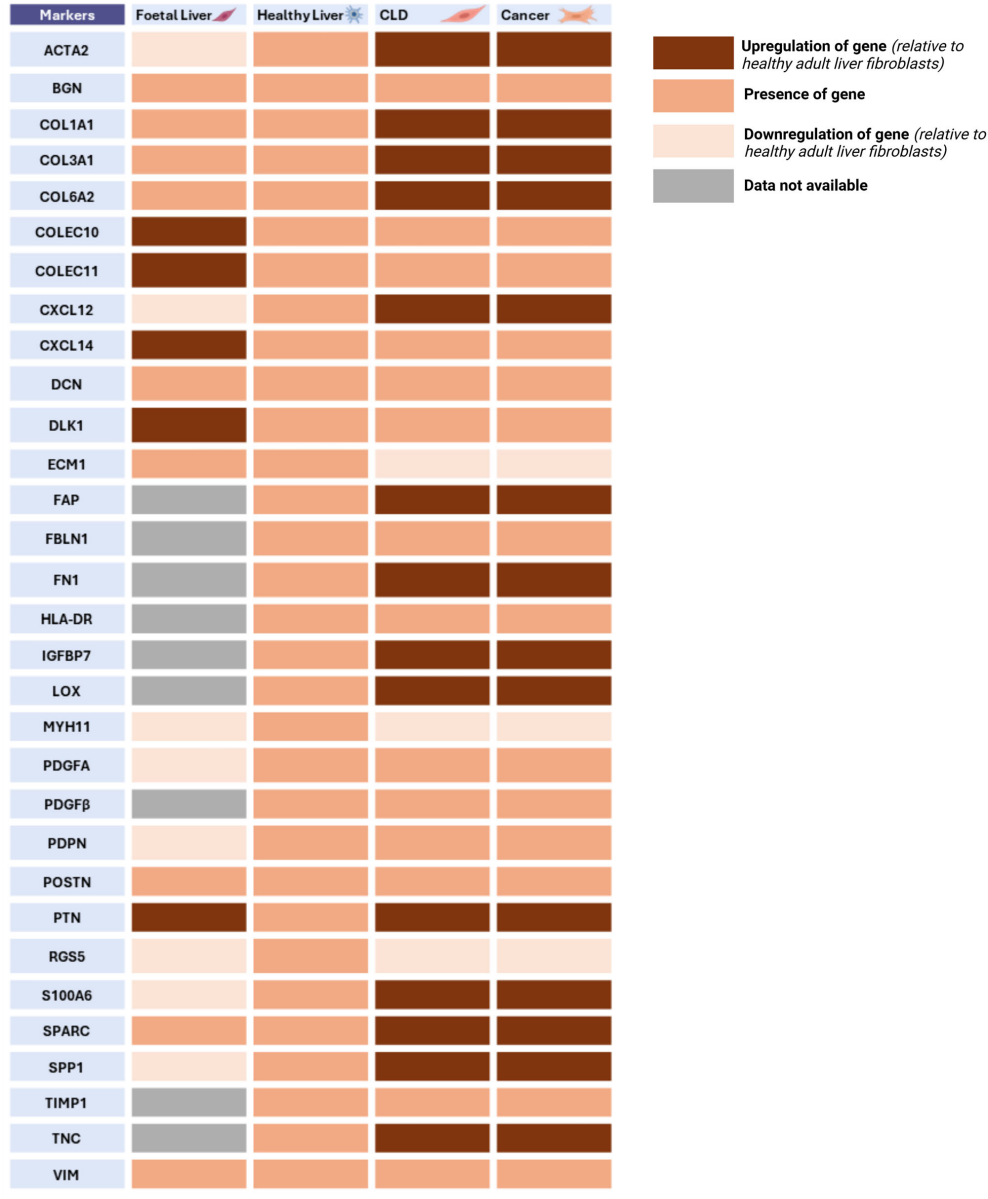
Comparative gene expression of hepatic fibroblasts from development to disease. Expression of selected fibroblast genes in human foetal to diseased liver (CLD), including cancer, based on publicly available scRNA-seq data. Genes were selected focusing mainly on ECM and other genes used for fibroblast classification across studies. Upregulation and down regulation were determined based on the reported analyses within each individual study as relative expression to fibroblasts derived from healthy liver.

This review highlights the multifaceted nature of these cells through recent findings obtained with sc/snRNA-seq technologies, which have revolutionised our understanding of their origin, function, and pathological transformation. Based on the scRNA-seq studies showing specific analyses of fibroblast markers and populations in the liver reported here, we have conducted a comparative summary of the reported gene expression profiles in fibroblasts across the stages from fetal to adult, focusing mainly on ECM and other genes used for fibroblast classification across studies (Fig. 4). Due to differences in study design, sample processing, and analytical approaches between datasets, the figure is intended to provide a comparative qualitative overview of reported expression patterns.

Genes such as *CXCL12* and *PTN* were shown to be more strongly expressed in foetal HSCs compared to healthy adult HSCs^23^. These genes were also differentially expressed between different stages of foetal liver development (from 5 to 19pcw), suggesting that they could play an important role in contributing to liver development during this timeframe. Interestingly, in this time window, HSPCs migrate and expand in the foetal liver; understanding the role of these genes and their upregulation in this period would be highly valuable in further understanding the role of HSCs in promoting HSPCs expansion during development^10^. Of note, PTN is a heparin-binding growth factor known to regulate growth and is involved in fate decisions of various stem and progenitor cells in the foetal liver niche. Previous mouse-model studies have shown that PTN promotes hepatocyte proliferation and is induced in regenerative conditions such as bile duct ligation and partial hepatectomy^122–124^. However, PTN has also been correlated to pathological processes: it is upregulated in liver fibrosis and various cancers including HCC^118,125,126^. Park et al. found that *PTN* is expressed by HSCs in the fibrotic liver, where it inhibits TGFβ1-induced apoptosis, thereby driving fibrinogenesis and tumorigenesis^118^. Similarly, Lin et al. identified *PTN* in CAF-2 as a key modulator in the progression from HBV-related cirrhosis to HCC, underscoring its clinical significance. This shows that scRNA-seq not only enables the identification of genes such as *PTN* in specific cell populations (foetal fibroblasts or disease-state fibroblasts), but it also helps tracking how their expression affects cellular states, whether by supporting hepatocyte growth during development or supporting tumour growth and fibrotic disease progression^117^. This emphasises the need to perform more comparative analysis of foetal liver mesenchymal populations over different stages alongside the adult liver to fully elucidate gene dynamics and cell populations during development.

Among ECM-related genes, *ECM1* was found to be upregulated in both foetal^10^ and healthy adult HSCs compared with CLD HSCs^59^. Link et al. reported that *ECM1* expression is enriched in quiescent HSCs compared to their activated state, implying their role in maintaining quiescence and function^127^. To follow, *ECM1* was shown to be downregulated in models of liver fibrosis, with their downregulation in hepatocytes leading to activated TGF*β*1 in the matrix to facilitate HSC activation^128^. Consistent with this, the same group revealed that ECM1 is a critical regulator in MASH^129^. Hence, investigating ECM1 regulation in fibroblasts could provide valuable insight into how its loss contributes to disease progression and fibrosis.

Definitive evidence for transitional human cell clusters bridging distinct hepatic fibroblast populations (e.g., periductal, periportal etc.) and classical HSCs remains elusive and represents an unresolved question in the field. RNA sequencing has unequivocally demonstrated robust heterogeneity within mesenchymal compartments of the liver, delineating both HSC subpopulations and multiple activated myofibroblast states with divergent expression profiles of ECM genes. However, these studies predominantly reveal distinct clustering of HSCs and myofibroblasts along activation or injury response trajectories, without directly identifying or comparing clusters that represent a continuous transcriptional transition between anatomically and molecularly defined fibroblasts and HSC lineages. For example, detailed HSC atlases integrate quiescent, initiatory, and activated myofibroblast states in disease, but these represent progression within the HSC lineage rather than a bridge from distinct fibroblast populations^59^. Concurrently, human scRNA-seq studies predominantly focus on state transitions within the HSC lineage itself rather than on cross-lineage plasticity with other fibroblast clusters. The human data have not yet revealed stable clusters with mixed fibroblast–stellate identities or clear pseudotemporal paths with transitional intermediates that share coherent expression programs with both fibroblast and HSC features. As a result, the question of whether these lineages can interconvert, or whether discrete transcriptional bridges exist in vivo, remains an important and unresolved issue. This gap matters because understanding potential lineage plasticity or cross-lineage transitional states could critically inform mechanistic models of fibrogenesis and therapeutic targeting, particularly in conditions where multiple mesenchymal sources contribute to myofibroblast pools.

Although single-cell RNA sequencing has significantly advanced our understanding of distinct fibroblast subtypes across various stages of liver development and disease, there remains a critical gap in comparative analyses. In adult liver scRNA-seq studies, current efforts have primarily focused on comparisons between healthy and diseased fibroblasts, or normal fibroblasts and CAFs. However, direct comparisons between fibroblasts derived from CLD and HCC/CCA-CAFs are largely missing. For example, both *COL1A1* and *FAP* are known to be upregulated in fibroblasts in MASH and HCC tissue. Yet there is no clear evidence indicating whether their expression levels further increase from the MASH disease state to HCC. Given that HCC commonly develops from a background of CLD such as MASH, it would be important to define whether fibroblasts undergo additional transcriptional reprogramming during this transitional state. A comparative analysis between the two can reveal whether CAFs not only retain CLD-like markers but if they acquire a more activated phenotype. Moreover, recently, the development of a small-molecule FAP inhibitor, AZD-2389, has been studied as a candidate therapeutic for MASH^130^. This further underscores the urgency of performing comparative analysis, as it could have direct clinical implications in identifying fibroblast’s precursor populations that could prevent HCC arising from MASH. Lineage tracing and fate-mapping combined with single-cell and spatial transcriptomics provide the most direct evidence for CAF origins and their relative contributions^131,132^. To definitively resolve CAF origins in the liver, future studies should integrate sc/snRNA-seq of paired human samples spanning from normal liver, CLD, dysplastic nodules, and established HCC/CCA. Integrating this data with sc-ATAC sequence could map development trajectories and define the regulatory mechanisms that drive cell fate decisions during differentiation. This could then be validated in vitro using tumour organoid co-culture models with lineage-restricted liver fibroblast populations and independently introduced to determine which cell type differentiates into the different CAF states.

RNA sequencing data can provide a foundational blueprint for constructing lineage-informed organoids and co-culture systems that capture microenvironment-specific interactions. This for example, could include modelling RSPO3–LGR4/5-mediated fibroblast-hepatoblast signalling, cell-ECM interaction, or mimicking CAF differentiation via the SPP1–CD44–AKT axis. Integrating in vitro experiments with scRNA-seq analysis, spatial mapping, lineage tracing, and functional validation will be critical in exploring the therapeutic potential of disrupting key fibroblast-mediated signaling pathways in liver disease and advancing precision therapies. To consider the functional implications of scRNA-seq-generated fibroblast profiles, the field should explore the matrix deposition and remodelling properties and secretome of identified HSC/fibroblast subtypes. Transcriptomics profiling studies produce important definitions of cellular subsets in development, homeostasis, and disease, and this evidence is useful to design progressively more complex and relevant in vitro systems. These models can be tailored to include specific subsets/populations and used to characterise their matrix remodelling activity, for drug discovery and testing^133^.

The accumulation of scRNA-seq data provides a major advantage in identifying markers that define specific fibroblast populations, enabling more precise targeting for further investigation. Markers such as *PTN*, *PRELP*, and *POSTN* offer promise for fibroblast-specific cell isolation (and their incorporation into multicellular in vitro models), biomarker discovery and targeted delivery^115,118^. However, challenges remain in evaluating fibroblast subtypes due to selective isolation and in vitro culture conditions. For instance, fibroblasts cultured on plastic monolayers or synthetic hydrogels may expand preferentially, leading to redundancy of certain subtypes and reducing the diversity observed in vivo. Plastic cultures often homogenise fibroblast populations, often becoming more myofibroblast-like after a few passages, characterised by the expression of *aSMA*. Elyada et al. illustrated that PDAC iCAFs preserved in Matrigel convert into myCAFs when cultured in 2D with tumour organoid-conditioned medium, while apCAFs acquire myCAF markers under similar conditions^134^. Ultimately, scRNA-seq datasets can aid in isolation strategies to ensure that fibroblast subtypes are preserved in vitro.

Overall, scRNA-seq studies examining liver fibroblast populations across development, homeostasis, and disease provide important insights but are sometimes limited by small sample sizes and technical constraints. A key limitation is the tissue dissociation process, which can introduce bias because fibroblast subsets embedded within dense ECM may be underrepresented compared to more easily dissociated cell types. Optimised robust protocols, such as the use of collagenase and hyaluronidase to degrade stiff ECM, particularly in fibrotic livers and tumours, may help improve their capture. Additionally, overlapping transcriptional profiles between fibroblasts, hepatic stellate cells, and other mesenchymal populations make it difficult to clearly distinguish stromal subtypes using transcriptomic data alone, meaning identified clusters may not represent fully distinct biological populations.

scRNA-seq has also elucidated potential pathways relevant in fibrosis, allowing for new therapeutic strategies to be developed. Several pathways that are involved in fibrosis progression, including fibroblast-mediated pathways are currently being explored as potential therapeutic targets. Currently, anti-TGFβ therapy has shown some potential in animal models and clinical trials. As of 2025, 3 drugs (PR-PFD, Hydronidone and Montelukast) are in clinical trial as potential drugs that can be used for fibrosis treatment, with one of their proposed mechanisms of action focusing on TGFβ suppression to inhibit fibroblast activation, intending to reduce synthesis of collagen type I and III. In 35.2% of patients in Phase II clinical trials of PR-RFD partial hepatic fibrosis reversal was observed, as assessed by a fibrosis regression profile^135^. A PDGFR tyrosine kinase inhibitor, Imatinib mesylate, underwent Phase 1 clinical trial in 2022 in Tehran, as a promising molecular approach to limit liver fibrosis via upregulation of miR-124 which simultaneously interferes with the STAT3/IL-6 pathway however, results are yet to be published^136^.LOXL1 inhibitors have also been explored in animal models, with liver fibrosis arrest observed in mice following LOXL1 inhibition, however no clinical trials are currently underway. FGF-mediated pathways are also being explored, with clinical trials ongoing for Aldaferrin (FGF19 analog), Pegozafermin (FGF21 analogue), Efruxifermin (FGF21 analogue)and Pegbelfermin (FGF21 analogue), all targeting the FGF signalling pathways in fibrosis with promising results^137^. Previously developed for other cancers including colorectal, pancreatic ovarian, and lung cases, FAP-targeting therapies, such as an anti-FAP antibody^138^ and a bispecific FAP-CD40 antibody^139^ have showed potential in increasing T cell infiltration and controlling tumour growth in preclinical models. These CAF-FAP targeting pathways could provide significant impact for use as an adjunctive treatment alongside HCC immunotherapy.

## Conclusions

While the ontogeny of hepatic fibroblasts and HSCs remains a subject of active investigation, recent compelling evidence supports a mesodermal origin, with contributions from septum transversum-derived mesothelial and submesothelial cells expressing *WT1* during development^18^. Functionally, HSCs and fibroblasts contribute to the haematopoietic niche during foetal development, producing cytokines such as FLT3L, CSF1, and CXCL12 that support HSPC expansion^11,34,35^. Additionally, HSCs and fibroblasts influence hepatoblast differentiation, hepatocyte function, and vasculature patterning through the secretion of key signalling molecules, including TGFβ, HGF, and VEGF, as well as ECM proteins^8,23^. ECM proteins such as collagens (types I, III, IV), fibronectin, laminin (for example, Laminin-521), elastin, and other glycoproteins and proteoglycans, are not just structural proteins but they actively participate in signalling pathways that guide hepatocyte fate. For instance, fibronectin and laminin interact with integrins expressed by hepatoblasts, triggering intracellular cascades that promote differentiation. Similarly, collagen IV and laminin help establish the basement membrane, which is essential for hepatocyte polarity and bile canaliculi formation. The interaction between HSCs/fibroblasts and hepatoblasts via the RSPO3–LGR4/5 axis further illustrates their role in shaping the hepatic niche and architecture^15,140,141^. This evidence highlights the need in the field to link fibroblast profiles with functional and proteomic analyses to identify the stages and cellular subtypes that support haematopoiesis and hepatogenesis.

In the adult liver, scRNA and snRNA sequencing has unveiled the transcriptional heterogeneity of hepatic fibroblasts and HSCs at homeostasis and during fibrosis progression. Studies have identified multiple subpopulations, including quiescent, pre-activated, and fully activated HSCs, as well as distinct mesenchymal clusters with varying degrees of fibrogenic potential^58,83^. These findings challenge the traditional binary view of HSCs as either quiescent or activated and suggest a spectrum of activation states influenced by microenvironmental cues. Importantly, ECM gene expression has emerged as essential for distinguishing between HSC populations and phenotypes. Genes such as *COL1A1*, *COL3A1*, *LOXL1*, *FN1*, *SPARC*, and *POSTN* not only stratify populations, but actively regulate cell fate, microenvironmental remodelling, and immune cross-talk^142^. RNA sequencing technologies have also revealed spatial and functional compartmentalisation within the liver. For example, scar-associated mesenchymal cells (SAMes) and cytokine-producing HSCs (cyHSCs) exhibit distinct gene expression profiles, immunomodulatory properties, and localisations, suggesting specialised roles and immune modulation in fibrosis^82^. Comparative scRNA-seq studies also reveal transcriptional parallels among HSC subpopulations across cirrhotic vs healthy livers. For example, *COL1A1/LOXL1*-enriched clusters^58,84^ and antigen-presenting HSC subsets^81^ demonstrate conserved fibrogenic and immunomodulatory profiles^142^.

**Fig. 5:**
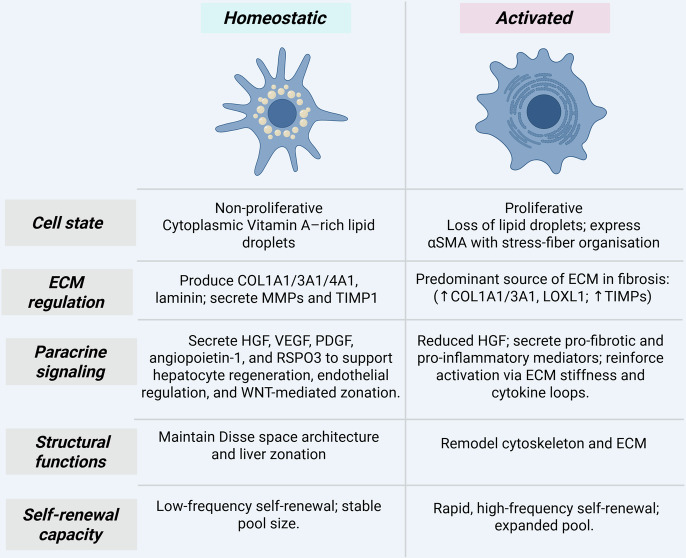
Phenotypic and metabolic transitions of hepatic stellate cells from homeostasis to activation. Created with BioRender.

Given the evidence of different functional activities in quiescent HSCs and fibroblasts, this study overturns the long-standing view of HSCs as passive or solely fibrogenic. Their transcriptomic profile includes specific genes involved in the matrisome, inflammation, and metabolism. Single-cell atlases describe HSCs in a “resting” or “baseline” cellular state beyond the strict concept of quiescence. These cells are functionally active even though they are in homeostasis rather than “activated” in response to a disease or injury The field may consider adopting another definition for HSCs to move away from “quiescent”, for example using “homeostatic” or “maintenance” HSCs as more general, neutral descriptor of normal function in the absence of injury or stimulation, describing a functional role in homeostasis, including maintain ECM composition and metabolic activities as outlined in Fig. 5. The significant overlap in markers challenges the current definition of quiescent HSCs and fibroblasts in homeostasis, suggesting that a different nomenclature may be considered to better describe these cells in their steady state.

In the context of primary liver cancers, CAFs promote tumour progression by remodelling the ECM, suppressing immune responses, and facilitating angiogenesis^95,112^. Studies reviewed here have shown distinct CAF subtypes, such as iCAFs, myofibroblastic CAFs (myCAFs), and CD36^+^ CAFs, each with unique transcriptional signatures and functional roles^106^. While in-depth analyses have revealed the origins of CAF subsets in HCC, there is still an open question as to the primary origins of CAFs in CCA. In the TME, pseudotime analyses have confirmed that CAFs emerge early in tumour development, preceding immune infiltration, and evolve into distinct subtypes with specialised functions^117^. Notably, CAFs also contribute to tumour immune evasion and angiogenesis through the secretion of immunomodulatory cytokines and ECM-remodelling enzymes, key drivers of HCC progression^88,143^.

Further insights on HSCs and fibroblast biology in the liver, from development to adulthood and disease, obtained via developing single-cell atlases, can have profound implications for understanding liver function and dysfunction, and for developing targeted therapies aimed at modulating fibroblast activity in pathological conditions.

## Data Availability

No datasets were generated or analysed during the current study.
